# Caspase-8 activation by TRAIL monotherapy predicts responses to IAPi and TRAIL combination treatment in breast cancer cell lines

**DOI:** 10.1038/cddis.2015.234

**Published:** 2015-10-01

**Authors:** R Polanski, J Vincent, U M Polanska, T Petreus, E K Y Tang

**Affiliations:** 1 AstraZeneca, Oncology Bioscience iMed, Alderley Park, Macclesfield, UK

## Abstract

The discovery of cancer cell-selective tumour necrosis factor-related apoptosis inducing ligand (TRAIL)-induced apoptosis generated broad excitement and development of TRAIL receptor agonists (TRA) as potential cancer therapy. Studies demonstrating the synergistic combination effect of SMAC mimetics and TRA further suggested potentially effective treatment in multiple tumour settings. However, predictive biomarkers allowing identification of patients that could respond to treatment are lacking. Here, we described a high throughput combination screen conducted across a panel of 31 breast cancer cell lines in which we observed highly synergistic activity between TRAIL and the inhibitors of apoptosis proteins (IAP) inhibitor (IAPi) AZD5582 in ~30% of cell lines. We detected no difference in the expression levels of the IAPi or TRAIL-targeted proteins or common modulators of the apoptotic pathway between the sensitive and resistant cell lines. Synergistic combination effect of AZD5582 and TRAIL correlated with sensitivity to TRAIL, but not to AZD5582 as a single agent. TRAIL treatment led to significantly greater activity of Caspase-8 in sensitive than in resistant cell lines (*P*=0.002). The majority (12/14) of AZD5582+TRAIL-resistant cell lines retained a functional cell death pathway, as they were sensitive to AZD5582+TNF*α* combination treatment. This suggested that failure of the TRAIL receptor complex to transduce the death signal to Caspase-8 underlies AZD5582+TRAIL resistance. We developed a 3D spheroid assay and demonstrated its suitability for the *ex vivo* analysis of the Caspase-8 activity as a predictive biomarker. Altogether, our study demonstrated a link between the functionality of the TRAIL receptor pathway and the synergistic activity of the IAPi+TRA combination treatment. It also provided a rationale for development of the Caspase-8 activity assay as a functional predictive biomarker that could allow better prediction of the response to IAPi+TRA-based therapies than the analysis of expression levels of protein biomarkers.

Induction of tumour-specific cell death is the most desirable effect of anticancer treatment.^1, 2^ Activation of death receptors expressed on tumour cells provides a selective way of inducing cell death and several lines of evidence suggest that therapeutic activation of death receptors such as TRAIL-R1 (tumour necrosis factor related apoptosis inducing ligand receptor) and TRAIL-R2 may provide the specificity to tumour cells^3^ with broad tolerability.^4^ Encouraging data has demonstrated anti-tumour activity of TRAIL receptor agonists (TRAs) in cell line-based preclinical models, in contrast to primary untransformed cells which show no significant response to TRAIL *in vitro*.^3^ However, a number of clinical trials demonstrated limited activity of TRAs as monotherapy and in combination with conventional chemotherapeutics, reviewed in.^1^ Resistance to TRAs could be attributed to a variety of mechanisms including increase expression of decoy receptors^5, 6^ or apoptosis modulators such as FLIP,^7^ inhibitors of apoptosis proteins (IAPs),^8^ antiapoptotic members of the BCL2 protein family,^9^ suppression of Caspase-8.^10^ A number of therapeutic strategies based on combination with small molecule inhibitors were proposed to unleash the potential of TRAIL receptors to induce tumour cell death.^1^ Synergistic activity of TRAIL with IAP inhibition^11^ and recently demonstrated CDK9 inhibitor^12^ are among the most exciting.

IAP is a family of eight antiapoptotic proteins in humans, sharing evolutionarily conserved Baculoviral IAP Repeat (BIR) domains.^13^ IAP are proto-oncogenes frequently overexpressed in cancers, which can potently inhibit apoptosis through different mechanisms including direct or indirect inhibition of caspases and activation of the NF-*κ*B pathway.^14^ Hence, IAPs are attractive therapeutic targets and at least five compounds have entered phase I/II clinical trials.^15^ The structure of IAP inhibitors is typically based on the similarity to the N-terminal part of Second Mitochondria-derived Activator of Caspase (SMAC), an endogenous inhibitor of IAP proteins;^16^ hence IAP inhibitors are also termed SMAC mimetics.

Synergistic activity of the combination has been demonstrated between SMAC mimetics and TRAIL in a number of tumour models and induces cell death in sensitive models;^1^ recently, a phase I clinical trial has been initiated to evaluate a combined regimen of a SMAC mimetic, birinapant, with an agonist monoclonal antibody against TRAIL-R2, conatumumab^15^ with anticipation that improved clinical response will be observed with this combination than monotherapy TRA treatment.

Predicting response to agents targeting the apoptotic pathway has proven challenging due to a high degree of complexity and redundancy in cell death pathways.^17^ To our knowledge, there are no validated biomarkers predicting response to TRAs or IAP inhibitors. Moreover, even preclinical research does not offer a clear guidance on development of predictive biomarkers to TRAs;^1^ similarly, we are only beginning to appreciate the challenge of predicting sensitivity to IAP inhibitors.^18^ Furthermore, predicting the response to simultaneous interference with two different components of the apoptotic machinery (IAP and TRAIL receptors), presents additional challenge. This is at least in part because the mechanisms responsible for the death signal transduction and execution are still not completely understood in the context of the combination treatment.

Here, we focused our attention on identification of molecular biomarkers that define the difference between the breast cancer cell lines that are either sensitive or resistant to a combined IAPi (AZD5582) and TRAIL treatment. We performed a high throughput combination screen on a large panel of cell lines and searched for markers of sensitivity or resistance. We found no differential expression of target proteins, nor of the modulators of the apoptotic pathway tested in this study between the sensitive and resistant cell lines. However, we demonstrated that sensitivity to TRAIL, but not to AZD5582, correlated with synergistic response to the combination treatment, and that TRAIL treatment leads to a significant increase in Caspase-8 activity in sensitive, but not in resistant cell lines. We subsequently established a protocol for *ex vivo* Caspase-8 biomarker analysis and confirmed feasibility of this method. Altogether our results suggest that functional biomarkers such as Caspase-8 activity as readout of the functionality of the TRAIL receptor pathway may prove superior to the analysis of protein expression, in predicting the response to IAPi+TRA combination treatment.

## Results

### Sensitivity or resistance to IAPi+TRAIL is independent of the expression levels of key cell death modulators

We performed a combination screen in a panel of 31 breast cancer cell lines to evaluate the synergism between the IAP inhibitor AZD5582 and TRAIL. We observed a high variability of phenotypic response with no synergistic tumour cell lethality in 13 cell lines (synergy score <4), strongly synergistic response and indication of enhanced cell killing in 10 cell lines (synergy score >10) and 10 cell lines showing intermediate synergy (synergy score >4 and <10) with a median synergy score of 5.1 (Table 1). Additionally, we performed a screen in 16 colorectal cancer cell lines; in contrast to the breast cell line panel, the majority of the colorectal tumour cell panel responded synergistically with a median synergy score of 33.5 (Supplementary Table 1).

**Figure 1:**
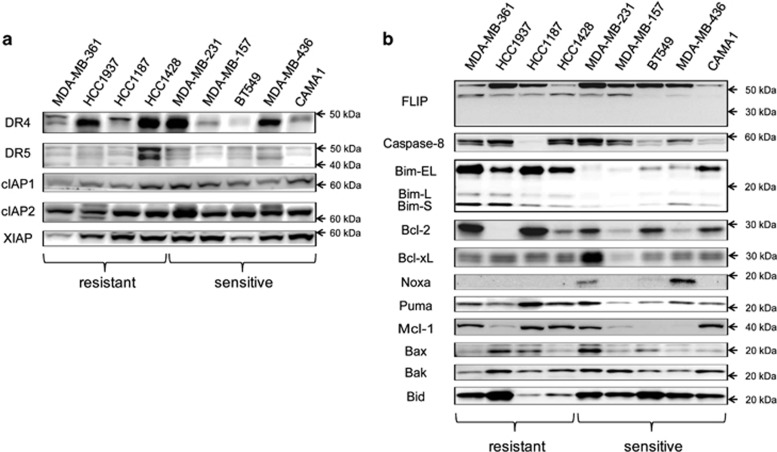
Expression of target proteins and other apoptotic factors does not correlate with the response to combined AZD5582 and TRAIL. Expression levels of target proteins (**a**) and key apoptotic regulators (**b**) were determined by western blotting in four AZD5582/TRAIL resistant and five sensitive cell lines. 20 *μ*g of protein lysate was used per lane for each cell line

We focused on the breast panel as it offered the opportunity to evaluate the potential determinant factors differentiating sensitive and resistant cell lines. First, we chose a subset of four completely resistant and five exquisitely sensitive cell lines to evaluate the protein expression levels of cIAP1, cIAP2 and XIAP (targets of AZD5582) and DR4 and DR5 (targets of TRAIL) (Figure 1a). We found that the corresponding target proteins were all expressed in all cell lines, although there was variability in their expression level. However, there was no statistically significant difference between the AZD5582+TRAIL-sensitive and -resistant cell lines (Supplementary Table S2). Subsequently, we examined the expression levels of key components of the apoptotic pathway, including FLIP, Caspase-8, Bim, Bcl-2, Bcl-xL, Noxa, Puma, Mcl-1, Bax, Bak and Bid (Figure 1b) but again failed to detect any significant differences between AZD5582+TRAIL-sensitive and -resistant cell lines (Supplementary Table S2). We also confirmed AZD5582 target engagement by monitoring degradation of cIAP1 resulting from autoubiquitylation and proteasomal degradation;^19^ reduction of cIAP1 was clearly observed at 2 and 8 h with 10 nM AZD5582 in both sensitive and resistant cell lines (Supplementary Figure S1).

### AZD5582+TRAIL-sensitive cells undergo rapid apoptosis following treatment, whereas resistant cells do not

**Figure 2:**
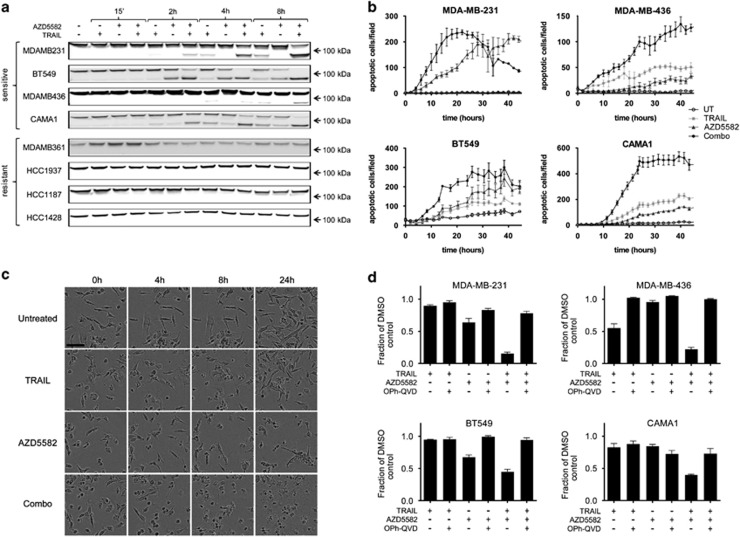
Combined AZD5582 and TRAIL synergize and induce caspase-dependent cell death in some breast cancer cell lines. (**a**) Four AZD5582/TRAIL-resistant and four sensitive cell lines, as indicated, were treated with 10 nM AZD5582, 10 ng/ml TRAIL or a combination for the indicated period of time and 20 *μ*g of protein lysate was used for each lane and PARP was detected by western blotting. (**b**) Caspase-activity was quantitated using Nuc-view visualised using Incucyte in four sensitive cell lines treated with 0.1 nM AZD5582 and 10 ng/ml TRAIL over the period of 45 h. Error bars represent S.E.M. of triplicates. (**c**) An example of cell morphology changes occurring to MDA-MB-231 cells in response to 0.1 nM AZD5582, 10 ng/ml TRAIL and a combination over the 24-h time course. (**d**) Cell viability was evaluated using proliferation-cell death assay following treatment with 0.1 nM AZD5582 and 10 ng/ml TRAIL in the presence and absence of a pan-caspase inhibitor OPh-QVD, over 24 h

To investigate the mode of action of the AZD5582+TRAIL treatment in our panel of cell lines, we examined cleavage of PARP by western blotting, as readout of apoptosis. As early as 2 h after treatment, we observed enhanced cleavage of PARP in response to the combination treatment, compared with single agents in three out of five sensitive cell lines, indicating the onset of apoptotic cell death (Figure 2a). Note that relatively high 10 nM concentration of AZD5582 was used in such short duration experiments to demonstrate the clear differential response between combination sensitive and resistant cell lines. We used 10 ng/ml TRAIL in all cell death assays which had limited effect in most cell lines (19% reduction in live cell number in MDA-MB-231, 12% in BT549, 41% in MDA-MB-436 and 21% in CAMA1 cells, data not shown). None of the resistant cell lines showed any evidence of PARP cleavage; loading controls are shown in Supplementary Figure S2A. In contrast, MDA-MB-157 cells exhibited a delayed effect of the combination treatment in comparison with other cell lines with clear signs of PARP cleavage becoming visible at 24 and 48 h (Supplementary Figure S2B). Time-dependent onset of Caspase-3/7 activity was also measured using fluorescent caspase substrate in four sensitive cell lines, which provided further evidence for early induction of apoptosis in response to combination treatment (Figure 2b). In addition, examination of MDA-MB-231 cell morphology following treatment with TRAIL, AZD5582 or combination revealed disintegration of cells into apoptotic bodies following combination treatment (Figure 2c). Finally, the pan-caspase inhibitor Q-VD-OPh was able to rescue the tumour cells from cell death induced by AZD5582+TRAIL (Figure 2d), confirming that sensitive cells were dying by caspase-dependent cell death in response to the combination treatment. Note that in cell death assays with later end point of 24 h (Figures 2c and d) and 45 h (Figure 2b), we used 100 pM concentration of AZD5582 that corresponded to inhibition not greater than at EC70 (30% reduction in live cell number) for any of the cell lines tested, as defined by the 24 h concentration response (Supplementary Figure S6).

### Synergy between AZD5582 and TRAIL correlates with sensitivity to TRAIL but not to TNF*α*

**Figure 3:**
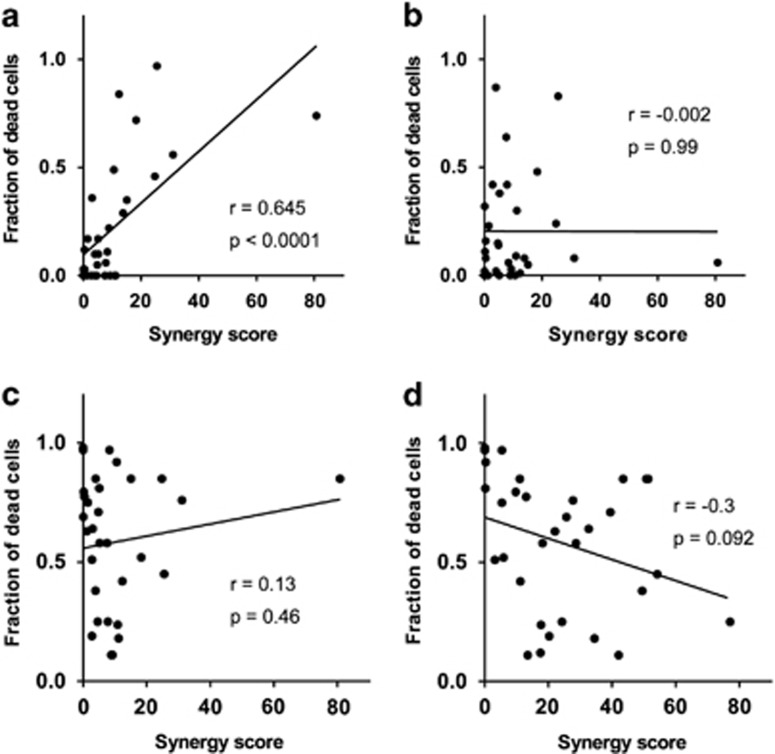
Synergy between AZD5582 and TRAIL correlates with response to TRAIL monotherapy but not to AZD5582 or TNF*α* monotherapy. (**a**) Correlation between TRAIL activity at 30 ng/ml concentration and synergy score of AZD5582/TRAIL combination treatment. (**b**) Correlation between AZD5582 activity at 10 nM concentration and synergy score of AZD5582/TRAIL combination treatment. (**c**) Correlation between TNF*α* activity at 30ng/ml concentration and synergy score of AZD5582/TRAIL combination treatment. (**d**) Correlation between TNF*α* activity at 30 ng/ml concentration and synergy score of AZD5582/TNF*α* combination treatment. Pearson's correlation coefficient (*r*) and *P*-values were calculated for each analysis

The breast tumour cell line panel responded variably to the IAPi+TRAIL combination treatment, illustrated by a broad range of combination synergy scores across the cell panel (Table 1). However, sensitivity to AZD5582 and TRAIL monotherapies was also highly variable (see heat maps in Supplementary Figure S4). To evaluate the contribution of each agent to the effect of combination synergy, we analysed our breast cancer cell line panel screening data and found that sensitivity to TRAIL as a single agent is a re-requisite and was strongly correlated with synergy of the combined AZD5582+TRAIL treatment, *P*<0.0001 (Figure 3a), however, sensitivity to AZD5582 did not, *P*=0.99 (Figure 3b). To gain further confidence that these results were TRAIL specific, we performed another screen where instead of TRAIL we used TNF*α* in combination with AZD5582. As for the combination with TRAIL, we also obtained a broad range of tumour cell lethality responses, but we observed no correlation between TNF*α* sensitivity and AZD5582+TRAIL combination response (Figure 3c); interestingly, there was also no correlation between sensitivity to TNF*α* and AZD5582+TNF*α* (Figure 3d), which is in contrast to the strong correlation between TRAIL sensitivity and AZD5582+TRAIL combination effect discussed previously (Figure 3a). Altogether these data suggested that signalling downstream of the TRAIL receptor had a key role in determining the synergistic response to the AZD5582+TRAIL in our cell line panel.

### Apoptotic signalling downstream from the death receptor is functional in most AZD5582+TRAIL-resistant cell lines and can be predicted by measurement of Caspase-8 activity

**Figure 4:**
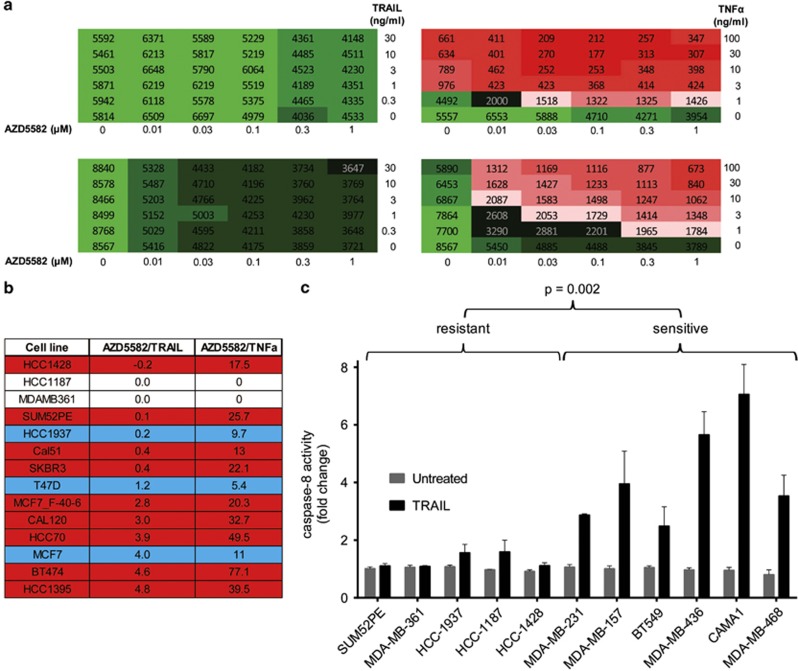
Extrinsic cell death pathway is functional downstream from Caspase-8 and defective upstream from Caspase-8 in the majority of AZD5582/TRAIL-resistant cell lines. (**a**) Heat maps showing examples of two cell lines where no synergy was observed between AZD5582 and TRAIL, but significant synergy was detected between AZD5582 and TNF*α*. (**b**) Summary of synergy scores obtained with AZD5582/TRAIL and AZD5582/TNF*α* combination treatments, blue shading indicates moderate difference and red highlights dramatic discrepancies between synergy scores obtained with the different treatment modalities. (**c**) Caspase-8 activity assay in cells treated for 16 h with 30 ng/ml TRAIL in five AZD5582/TRAIL resistant and six sensitive cell lines; error bars represent S.E.M. of means of three independent experiments

TNF*α* mediates its cellular effect through binding to one or more member of the TNF receptor super family, which recruit different adaptor proteins to elicit activation of Caspase-8 leading to a subsequent onset of apoptosis. To determine the functional lesion within the TRAIL signalling pathway in the resistant cells, we analysed the ability of AZD5582+TRAIL-resistant cell lines to respond to the AZD5582+TNF*α* treatment. Surprisingly, most of the AZD5582+TRAIL-resistant cell lines were exquisitely sensitive to the AZD5582+TNF*α* treatment (Figure 4a) and altogether 12 out of 14 most AZD5582+TRAIL-resistant cell lines were sensitised by the AZD5582+TNF*α* treatment (Figure 4b, Supplementary Figure S5). This suggests that the cell death pathway downstream from Caspase-8 is functional and the effects elicited by the two different combinations may be strikingly distinct from each other.

We further hypothesised that measurement of Caspase-8 activity following TRAIL treatment could predict sensitivity to AZD5582+TRAIL treatment. This was examined by measuring Caspase-8 activity in response to TRAIL in a panel of five resistant and six sensitive cell lines. We observed induction of Caspase-8 in all sensitive and only marginal increase in two resistant cell lines (Figure 4c) and the extent of Caspase-8 activation was significantly greater in magnitude in sensitive cell lines (*P*=0.002). These data are further supported by western blot analysis showing Caspase-8 cleavage in sensitive but not in resistant cell lines (Supplementary Figure S3) and suggest that (i) the death receptor-downstream death signal execution pathway is functional in the majority of AZD5582+TRAIL combination-resistant cell lines; (ii) the defect in signal transduction is upstream from Caspase-8 in resistant cells and (iii) this can be predicted by measuring Caspase-8 activity in TRAIL-treated cells.

### *Ex vivo* measurement of TRAIL-induced Caspase-8 activity demonstrates feasibility of the new biomarker approach

**Figure 5:**
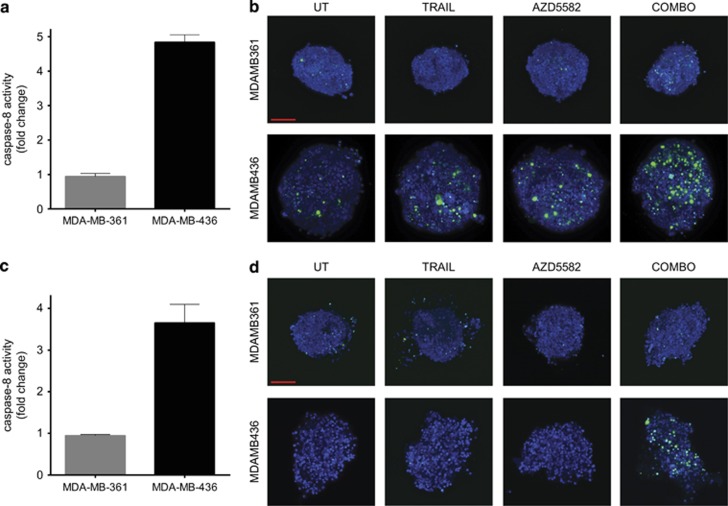
Demonstrating Caspase-8 activity assay as a predictive biomarker feasibility in spheroid cultures. Spheroids of MDA-MB-361 (AZD5582+TRAIL combination resistant) and MDA-MB-436 (combination sensitive) cells were treated with 30 ng/ml TRAIL followed by the Caspase-8 activity assay 16 h later (**a**) or treated with 10 ng/ml TRAIL, 10 nM AZD5582 or a combination for 24 h followed by fluorescence detection of Caspase3/7 activity. (**b**) Xenograft-derived spheroids of MDA-MB-361 (AZD5582+TRAIL combination resistant) and MDA-MB-436 (combination sensitive) models were treated with 30 ng/ml TRAIL followed by the Caspase-8 activity assay 16 h later (**c**) or treated with 10 ng/ml TRAIL, 10 nM AZD5582 or a combination for 24 h followed by fluorescence detection of Caspase-3/7 activity. (**d**) Error bars represent S.E.M. of means of three independent experiments (**a**) or independent experiments performed on tumour-derived spheroids from three different mice (**c**). Images in (**b**) and (**d**) were obtained using lightsheet microscopy and presented as maximum intensity projections of 200 *μ*m depth of the spheroid, Hoechst 33342 is blue, and Caspase3/7 signal is green. Scale bar represents 100 *μ*m

Having established that Caspase-8 activity following TRAIL treatment can discriminate between AZD5582+TRAIL combination sensitive and resistant cell lines, we considered this method as a potential predictive biomarker. Analysis of such a future functional biomarker would have to be performed in viable cells extracted from patients. Therefore, we used 3D spheroid cultures to demonstrate feasibility of our approach. First, we cultured MDA-MB-361 (combination resistant) and MDA-MB-436 (combination sensitive) cells as 3D spheroids and subsequently subjected them to TRAIL treatment followed by the Caspase-8 activity assay. Similarly to 2D, we detected no effect in MDA-MB-361, but we observed over four-fold increase in Caspase-8 activity in MDA-MB-436 cells (Figure 5a). We also performed fluorescent detection of Caspase-3/7 activity using lightsheet microscopy spheroids, which confirmed robust induction of apoptosis in response to the combination treatment in MDA-MB-436 but not in the MDA-MB-361 spheroid model (Figure 5b). Subsequently, we grew the MDA-MB-361 or MDA-MB-436 cells as xenografts in nude mice, harvested tumours, extracted cells, generated spheroids and subjected them to TRAIL treatment and the Caspase-8 activity assay. We detected no increase in Caspase-8 activity in MDA-MB-361 and almost four-fold induction in MDA-MB-436 (Figure 5c). We also observed robust induction of apoptosis in response to the combination treatment as defined by Caspase-3/7 activity visualised using lightsheet microscopy in MDA-MB-436 but not in MDA-MB361 *ex vivo* spheroids (Figure 5d). Altogether our results presented in Figure 5 suggest that it is feasible to perform Caspase-8 activity assay on the *ex vivo* material, which paves the way for future analysis of human patient samples.

## Discussion

Initial evaluation of TRAs in human preclinical cancer models provided hope for potentially well-tolerated, tumour-specific anti-cancer therapies. However, clinical trials of a number of different TRA agents proved generally disappointing with very few clinically significant responses in patients receiving the monotherapy or chemo-combination with the TRA agents.^1^ With the potentially well-tolerated characteristics of TRAs, a significant interest has been maintained to further develop TRA-based therapeutic approaches. In this context, two major advances are required to utilise TRAs in the treatment of cancer: highly synergistic combinations with other therapeutic agents and the development of suitable predictive biomarkers for patient stratification.

The spectrum of factors mediating resistance to TRAs is very broad and the blockade in the transduction of the death signal may occur at multiple levels of the apoptotic/cell death pathway, preventing the realisation of the clinical efficacy of TRAs. Epigenetic silencing, mutations and defective glycolysation of death receptors occurs frequently in TRAIL-resistant models; we initialised the study through profiling the target protein expression of AZD5582 and TRAIL and detected no statistically significant differences between sensitive and resistant cell lines in the breast tumour cell panel. This is consistent with reports that expression levels of TRAIL receptors are generally not predictive of the response to TRAs.^20^ This might be due to a multiplicity of other factors that determine response to TRAs. Among these, FLIP (Flice-like inhibitory protein) that functions as an inhibitor of Caspase-8 activation, overexpression and amplification of IAP, overexpression of anti-apoptotic and downregulation of proapoptotic members of Bcl2 protein family were shown to confer resistance.^21^ However, we observed no significant correlation between cell sensitivity and expression of key effectors and modulators of the cell death pathway. Some of the analysed proteins, the most notably proapoptotic Bim and Puma, displayed expression pattern that bordered on significance; however, their expression levels were higher in resistant cells that appear inconsistent as a resistance mechanism. On the other hand, expression levels of antiapoptotic MCL-1 were somewhat higher in resistant cell lines, however this was also not significant (*P*=0.07) and furthermore the direction of differential expression lacked clarity as some sensitive cells expressed increased and some resistant reduced levels of Mcl-1 (Figure 2b). These results further suggest that the analysis of these biomarkers may provide limited utility in predicting sensitivity to the IAPi+TRAIL combination treatment. Expression of Caspase-8 was also not significantly altered in resistant cell lines; this is in contrast with recently published data that identified Caspase-8 expression levels as a biomarker of TRAIL sensitivity in HNSCC,^22^ suggesting a potentially different mode of suppression of TRAIL receptor-dependent apoptosis in breast cancer. Despite lack of difference in TRAIL receptor or Caspase-8 expression between sensitive and resistance cells, we found that the AZD5582+TRAIL combination effect was strongly correlated with TRAIL sensitivity; we observed no synergistic AZ5582+TRAIL combination effect only in 1 (Cal120) out of 12 cell lines in which TRAIL as a monotherapy reduced cell viability by more than 20%.

Interestingly, the cell death pathway downstream of Caspase-8 appeared to be functional in AZD5582+TRAIL-resistant cell lines since the majority of these cells responded robustly to AZD5582+TNF*α*, suggesting a defect in TRAIL receptor-dependent activation of Caspase-8. Signalling from TRAIL to Caspase-8 could be blocked through several mechanisms, including receptor endocytosis,^23^ O-glycosylation,^24^ expression of Galectins^25^ or defective assembly of the DISC.^26^ To by-pass the need to confirm or exclude each of these resistance mechanisms, we measured Caspase-8 activity and found that it was substantially increased in AZD5582+TRAIL-sensitive cells compared with resistant cell lines following TRAIL treatment. This suggests that measurement of Caspase-8 activity in response to TRAIL treatment alone could serve as a functional predictive biomarker for the IAPi+TRA treatment. The added benefit of measuring Caspase-8 activity is the opportunity to specifically evaluate functionality of the TRAIL receptor pathway in response to TRAIL, which we show is associated with AZD5582+TRAIL synergy (Figure 3a). We observed that the apoptotic response to the AZD5582+TRAIL combination treatment is temporal and concentration dependent such that addition of AZD5582 not only enhances, but may significantly accelerate the onset of the TRAIL effect (Figures 2a and b). In the emerging trend of patient stratification, personal diagnostic will become increasingly critical for directing treatment regime. With limited amount of biopsy material available, detailed genetic and pharmacological evaluation of the primary material may not be feasible and, rapid measurement of Caspase-8 activity in primary cells derived from such samples in response to TRAIL may provide a solution. Until recently culture of primary tumour cells presented considerable challenge hence limiting the possibility of performing experiments using patient derived live cells not established as cell lines.^27^ With development of 3D tissue culture technologies such as culture of tumour slices^28^ or *ex vivo* culture of circulating tumour cells^29^ as well as more physiologically compatible media formulations this became more feasible.^30, 31, 32^ Functional diagnostic assays are already being developed for clinical use; for example, BH3 profiling that is a method for determining the potential of cells to effect the mitochondrial apoptotic pathway in response to treatment was shown to predict sensitivity to chemotherapy or the BH3 mimetic ABT-737 in cell lines^33^ and in clinical samples.^34^ Predicting combination effect of two or more agents is notoriously difficult,^35, 36^ and *in vitro* analysis of synergy is complicated (as described in Materials and methods). Therefore, our proposed approach based on measurements of Caspase-8 activity following TRAIL treatment has a potential to simplify the process as it is performed using a simple one-step assay following treatment with one concentration of TRAIL thus avoiding the need to measure synergy. It relies on our observations that (i) sensitivity to TRAIL as a single agent correlates with TRAIL+AZD5582 synergy, (ii) cell death pathway is generally functional downstream but defective upstream from Caspase-8 in combination resistant cell lines and (iii) functionality of the TRAIL receptor pathway can be probed and the combination effect predicted using the Caspase-8 activity assay. We also suggested an appropriate *in vitro* assay to allow Caspase-8 activity measurements, confimed feasibility, and optimised the work flow thus preparing a platform for future analysis of patient samples. It remains uncertain which are the key factors that determine the tumour cell sensitivity to AZD5582 as a single agent. Through the tumour cell panel profiling, we have observed a significant degree of sensitivity to the agent in a number (~30%) of cell lines (Supplementary Figure S4). Although it was previously reported that auto- and paracrine TRAIL and TNF*α* may sensitise the treated cells to SMAC mimetics,^37^ other mechanisms should not be ruled out. We hypothesise that AZD5582 sensitivity could result from high mitochondrial priming which is often the case in tumour cells,^38^ leading to high basal level of Bax/Bak activation. Whether IAP (which is often overexpressed in cancer) would in such cases prevent leaky activation of caspases, remains unclear, but this presents a compelling biomarker opportunity that should be explored further.

In conclusion, this study, as well as a large body of published evidence, suggests that analysis of basal expression of effector proteins as biomarkers may not be effective in predicting the response to IAPi+TRA treatment and instead functional assays might be required to achieve reliable predictions early in patient treatment. Development of organotypic tumour culture platforms, which is recently gaining significant traction, enables drug response prediction using patient-derived cells prepared from a biopsy material; this will likely shape future personalised cancer medicine.^39, 40, 41^ In this report, we describe the large-scale high throughput *in vitro* combination screen across a broad breast cancer cell line panel and identify significant subsets of either exquisitely sensitive or completely resistant cell lines to facilitate the study of sensitive and resistance mechanisms to specific treatment. Our data provide insight into the molecular determinants that govern the response to a combined treatment of the IAP inhibitor AZD5582 and TRAIL which allowed identification of Caspase-8 as a potential functional biomarker. Clinical trials evaluating IAPi+TRA combination treatment have recently been initiated, which warrants research towards further validation and development of this biomarker to address an urgent unmet clinical need and support the development of a potentially well-tolerated and effective anticancer treatment across multiple tumour types.

## Materials and Methods

### Cell culture

Cell lines were from ATCC except for and HT-29, Colo741, SW620, HCA-7, C32 and C75 (ECACC) were cultured in humidified incubators at 37 °C with 5% carbon dioxide. Cell lines and culture media used for propagation and *in vitro* experiments are listed in Supplementary Table S3. Cells were tested for mycoplasma and authenticated using DNA fingerprinting short-tandem repeat (STR) assays. Basal media RPMI-1640 phenol red-free, DMEM, Ham's F-12, McCoy's 5a, EMEM, IMDM, sodium pyruvate, L-glutamine, hydrocortisone, insulin and non-essential amino acids (NEAA) were from Sigma, Gillingham, UK; IMEM (zinc modified), glutamax and FCS were from Gibco, Paisley, UK. The high-throughput screen was performed for all cell lines using RPMI-1640 phenol red-free media supplemented with 10% FCS.

### Protein expression analysis

Cells cultured in 6-well plates were lysed on ice with a buffer containing 25 mmol/l Tris/HCl pH=6.8, 3 mmol/l EDTA, 3 mmol/l EGTA, 50 mmol/l NaF, 2 mmol/l sodium orthovanadate, 270 mmol/l sucrose, 10 mmol/l b-glycerophosphate, 5 mmol/l sodium pyrophosphate, and 0.5% Triton X-100 and protease and phosphatase inhibitor cocktails (Sigma). Protein concentration was measured using the BCA Assay (Thermo Scientific, Paisley, UK), lysates were diluted with sample loading buffer (Life Technologies, Paisley, UK) and 20 *μ*g of protein was loaded per well and separated on gradient 4–20% Bis-Tris Criterion gels (Bio-Rad, Hemel Hempstead, UK), transferred onto nitrocellulose membranes using the iBlot Dry Transfer System (Life Technologies), incubated with a blocking solution of 5% BSA in 0.05% TBST buffer and incubator overnight with primary antibodies (antibodies are listed in Supplementary Table S4) in 0.05% TBST, followed by 30 min washing in 0.05% TBST, and incubation with HRP-tagged secondary antibodies (Cell Signalling Technologies, New England Biolabs, Hitchin, UK) for 1 h and subsequent washing in 0.05% TBST for 30 min. Luminescence was detected with Syngene ChemiGenius using Super-Signal West Dura Chemiluminescence Substrate (Thermo Scientific).

### 384-well format proliferation-cell death assay and high throughput screen

For the high throughput screen, 500–2500 cells were seeded per well (the list of seeding density for individual cell lines is in Supplementary Table S5) of a 384-well Griener black clear bottomed tissue culture treated plate using Multidrop Combi (Thermo Scientific) and incubated overnight to allow cell attachment. A day 0 plate was set up to define the baseline cell number; this plate was assayed at the time of treatment. The following day cells were treated with indicated compounds using acoustic dispenser Echo 555 (Labcyte, Sunnyvale, CA, USA) and cultivated for 5 days. The dead cell assay was developed by adding 5 *μ*l of 2 *μ*mol/l Sytox Green (Life Technologies) in TBS/5 mmol/l EDTA to each well of the 384-well plates followed by incubation for 1 h at room temperature and reading on the Acumen. Subsequently, 10 *μ*l of 0.25% saponin in TBS/5mM EDTA was added to each well, incubated at room temperature for 16 h and re-read using the Acumen as previously for the dead cells read.

Heatmaps were generated for visual purposes. The live cell count was determined by subtracting the dead cell count from the total cell count. These data were then processed using an MS Excel macro to generate heatmaps of live cell count compared with day 0 values. The macro applied conditional formatting to the live values as a two colour scale—values below day 0, which indicate cell death, are coloured on a red scale and values above day 0 indicating anti-proliferative effects are coloured on a green scale. The day 0 value was used as the point at which the colour scales change. This produced a visual representation of the data and allowed patterns of synergy to be detected as inhibition effects greater than monotherapy alone.

Synergy scores were generated using the Genedata Screener software package combinations module to generate synergy scores using the Loewe synergy model. The synergy scores are heavily weighted for cell death effects by the combined compounds to allow more effective ranking of the combination. Synergy scores are calculated against the full matrix (6 × 6) of data and take into consideration all the concentrations tested. The package determines the deviation of the data away from a predicted model of additivity based on the monotherapy values and assigns a synergy score according to the level of ‘excess'.

### Spheroid culture and tumour extraction

Cells were harvested by trypsinisation, counted and 1000 cells was seeded per one well of a 96-well round bottom clear cell repellent plate (Greiner, Stroudwater, UK) in normal culture media supplemented with 1% matrigel. Culture plates were subsequently centrifuged at 300 × *g* for 5 min to bring all cells into proximity at the bottom of the ‘U'-shaped well. Spheroids formed were ready for experiments within 3–4 days.

Cells were extracted from xenografts by incubation of small tumour fragments generated by cutting with a scalpel with a mix of 1 mg/ml DNase (Qiagen), 120 *μ*g/ml collagenase (Sigma) and 500 *μ*g/ml dispase (Sigma) in serum-free media at 37 °C for 1 h with gentle rocking. Subsequently, cells were sieved through 70 *μ*m filter, counted and 1000 cells was seeded per one well of a 96-well round bottom clear cell repellent plate (Greiner) in serum and phenol red-free Mammary Epithelial Cell Basal Medium, Promocell (xenograft-extracted tumour cells failed to form spheroids and died in normal media) supplemented with 1% matrigel. Culture plates were subsequently centrifuged at 300 × *g* for 5 min. Spheroids formed were ready for subsequent experiments within 3–4 days.

### Sample preparation and lightsheet microscopy

Spheroids were treated with 5 *μ*M Cellevent Caspase-3/7 reagent (Life Technologies) for 1 h at 37 °C followed by fixation with 4% PFE and staining with 1 *μ*M Hoechst 33342 for 1 week at 4 °C. For the purpose of lightsheet microscopy (Zeiss Lightsheet Z.1) spheroids were suspended in 1% low temperature melting point agarose (Sigma), aspirated into a 2-mm-thick glass capillary insertable into the imaging chamber of the light sheet. In all, 488 and 405 nm lasers were used to excite green and blue fluorescence, respectively, the cube filter included a Laser Block Filter (LBF) 405/488/561 nm and a long-pass 490 nm dichroic mirror. Acquisition mode was set to 16 bit image, dual side fusion system, frame size 678 × 678 *μ*m and pixel size 0.35 *μ*m. Imaging was through the agarose core withdrawn from the capillary; Z-stack was performed to a depth of 200 *μ*m of the spheroid and sectioned every 1 *μ*m.

Images were further processed in ZEN2014 software to display the maximum intensity projection for the z-stack multidimensional data set from spheroids. Maximum intensity projection confers the output image with the pixels that contain the maximum value over all images in a stack (a 3D construct) for a specific pixel location. All images were processed following the same protocol.

### Incucyte

Real-time evaluation of apoptotic cell death was performed using the Cellplayer kinetic Caspase-3/7 assay reagent (Essen Bioscience, Welwyn Garden City, UK) in cells subject to siRNA knockdown and compound treatment in 384-well Griener black clear bottomed tissue culture treated plate, imaged with Incucyte Zoom under × 20 objective for 72 h (Essen Bioscience).

### Compounds and kits

IAP inhibitor AZD5582 was described before,^19^ recombinant human TRAIL and the pan-caspase inhibitor Q-VD-OPh were from Sigma, and Caspase-8 Glo detection kit was from Promega, Southampton, UK.

## Figures and Tables

**Table 1:** AZD5582+TRAIL synergy scores across the panel of breast cancer cell lines

**Cell line**	**AZD5582+TRAIL synergy score**
HCC1428	−0.2
HCC1187	0
MDAMB361	0
SUM52PE	0.1
HCC1937	0.2
Cal51	0.4
SKBR3	0.4
T47D	1.2
CAL120	3.0
HCC70	3.9
MCF7	4
BT474	4.6
HCC1395	4.8
MFM223	5.1
HCC38	5.2
Cal148	7.5
MCF7751_T17	7.8
KPL4	8.3
MCF7_F-100-3	8.9
DU4475	9.3
HCC1806	10.6
ZR751	10.9
MDAMB453	11.2
BT20	12.4
Sw527	13.8
MDAMB436	15.1
MDAMB157	18.3
MDAMB468	24.8
CAMA1	25.5
BT549	31.1
MDAMB231	80.8

## Abbreviations

BIR: Baculoviral IAP Repeat
DR: death receptor
IAP: inhibitors of apoptosis proteins
PARP: Poly (ADP-Ribose) polymerase
SMAC: second mitochondria-derived activator of caspases
TNF: tumour necrosis factor
TRAIL: TNF-related apoptosis-inducing ligand
TRA: TRAIL receptor agonist
