# Supplementary material for: Caspase-8 activation by TRAIL monotherapy predicts responses to IAPi and TRAIL combination treatment in breast cancer cell lines
Source: Cell Death Dis. 2015 Oct 1;6(10):e1893–. doi: 10.1038/cddis.2015.234 (PMC4632282; doi:10.1038/cddis.2015.234)
Supplement: Supplementary Information [file cddis2015234x1.docx]

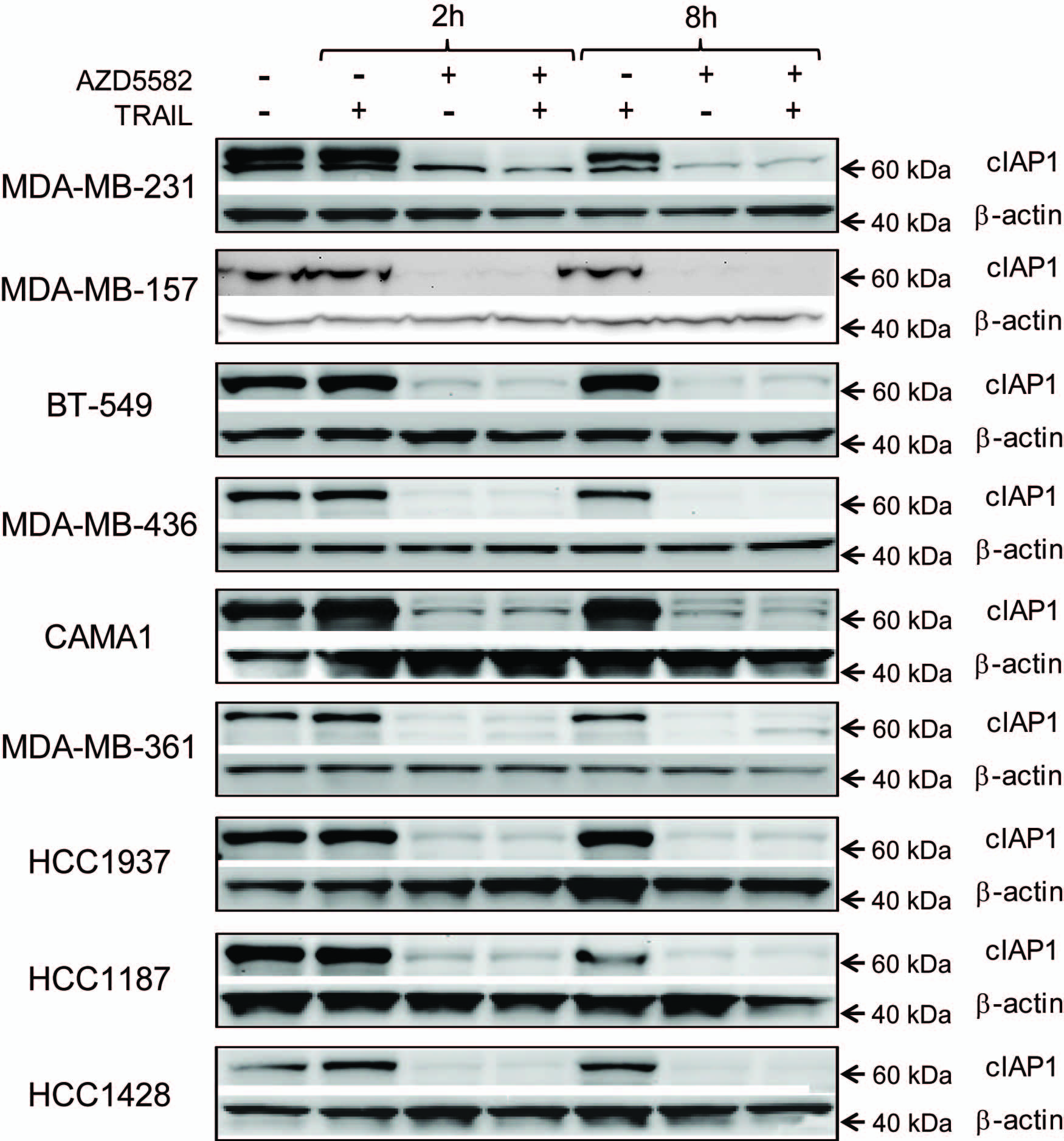


**Supplementary Figure S1. Confirmation of AZD5582 target engagement.** Cells were treated with 10ng/ml of TRAIL, 10nM AZD5582 or a combination and harvested at 2h and 8h. 20µg of protein lysate was used for each lane and expression of cIAP1 was probed on a western blot.


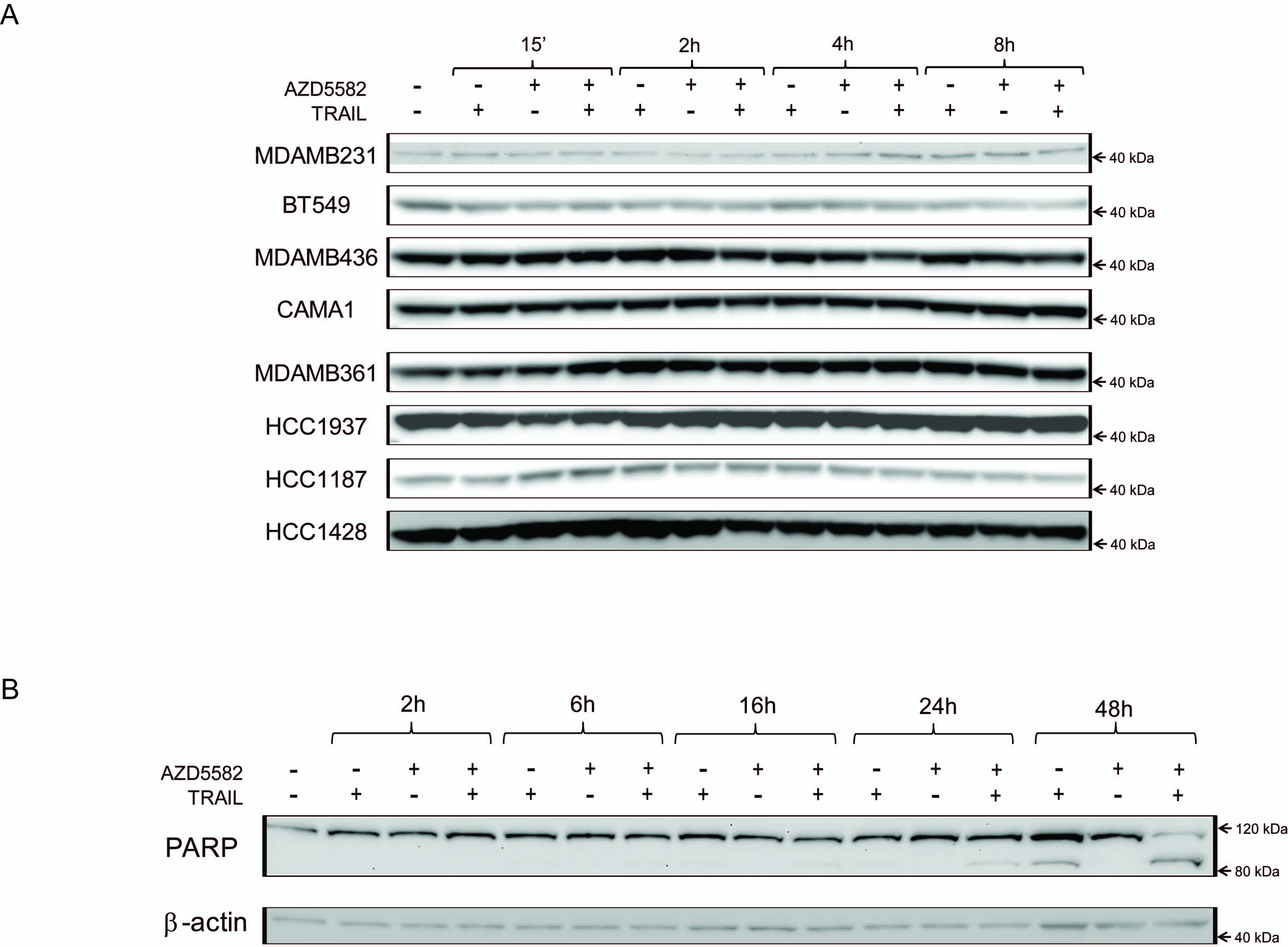


**Supplementary Figure S2. A. Loading controls for Figure 2A, B.** An AZD5582/TRAIL sensitive cell line MDA-MB-157 was treated with 10nM AZD5582, 10µg/ml TRAIL or a combination for the indicated period of time and 20µg of protein lysate was used for each and PARP was detected by western blotting.


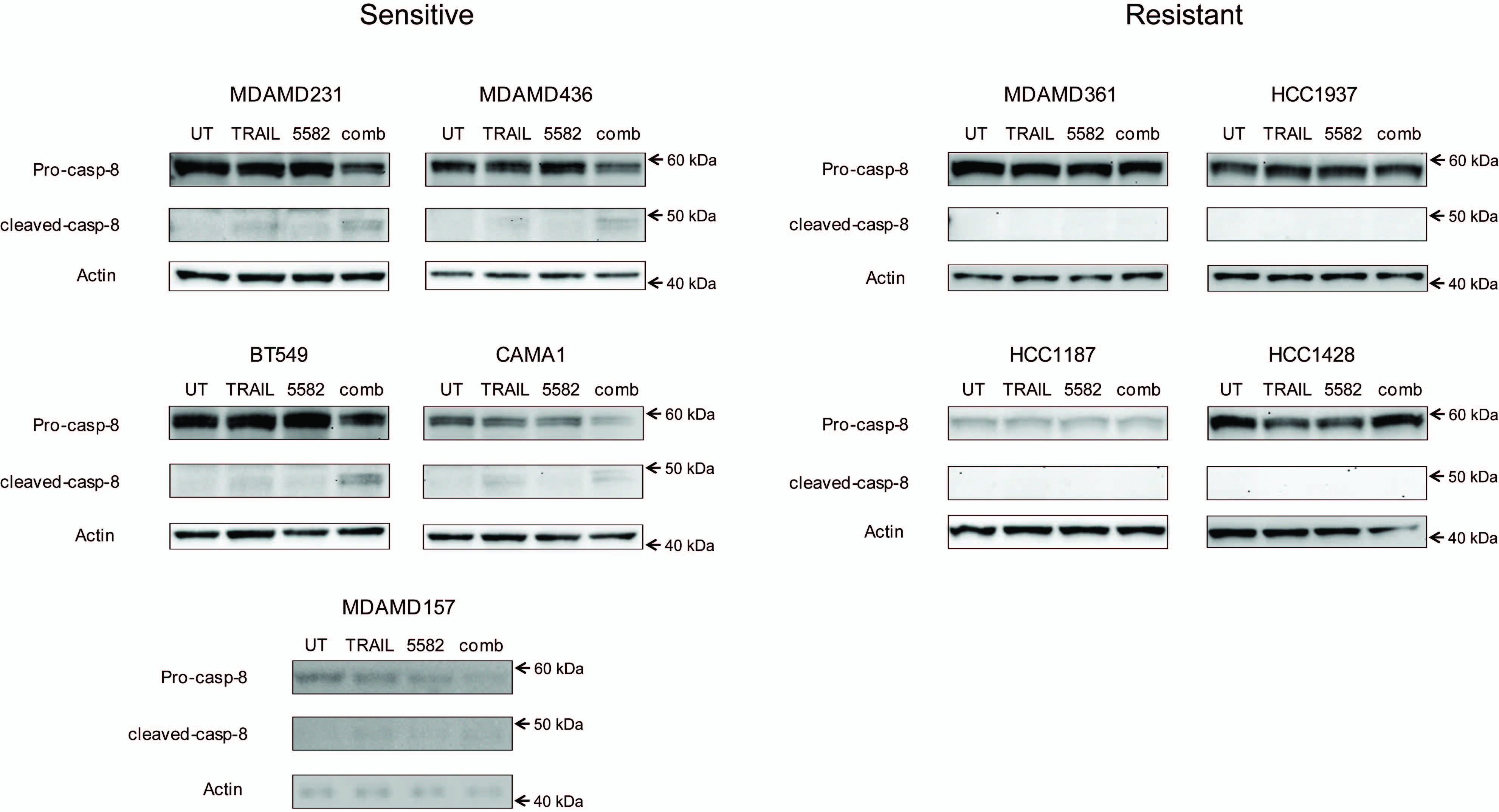


**Supplementary Figure S3. Cleavage of Caspase-8.** Four AZD5582/TRAIL resistant and four sensitive cell lines, as indicated, were treated with 100pM AZD5582, 10ng/ml TRAIL or a combination for the indicated period of time and 20µg of protein lysate was used for each lane and Caspase-8 was detected by western blotting, ~55kDa top band corresponding to a full length protein, and 41/43kDa band corresponding to the cleaved intermediate. We used a significantly shorter exposure to visualize the main pro-caspase-8 band as cleavage products induced with 10ng/ml TRAIL appeared relatively faint. We failed to detect 18kDa band with the Santa Cruz Biotechnology C-20 antibody.


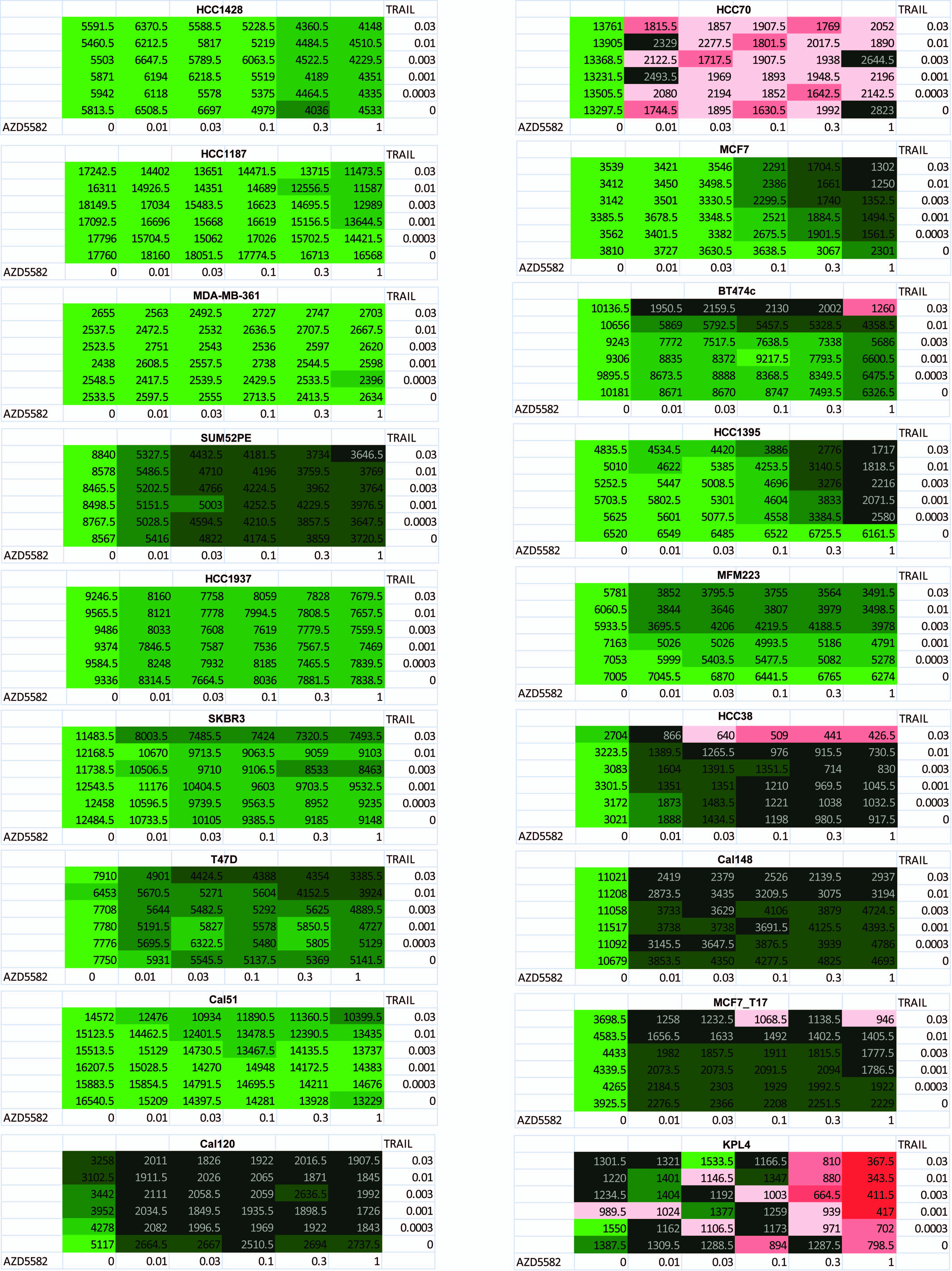


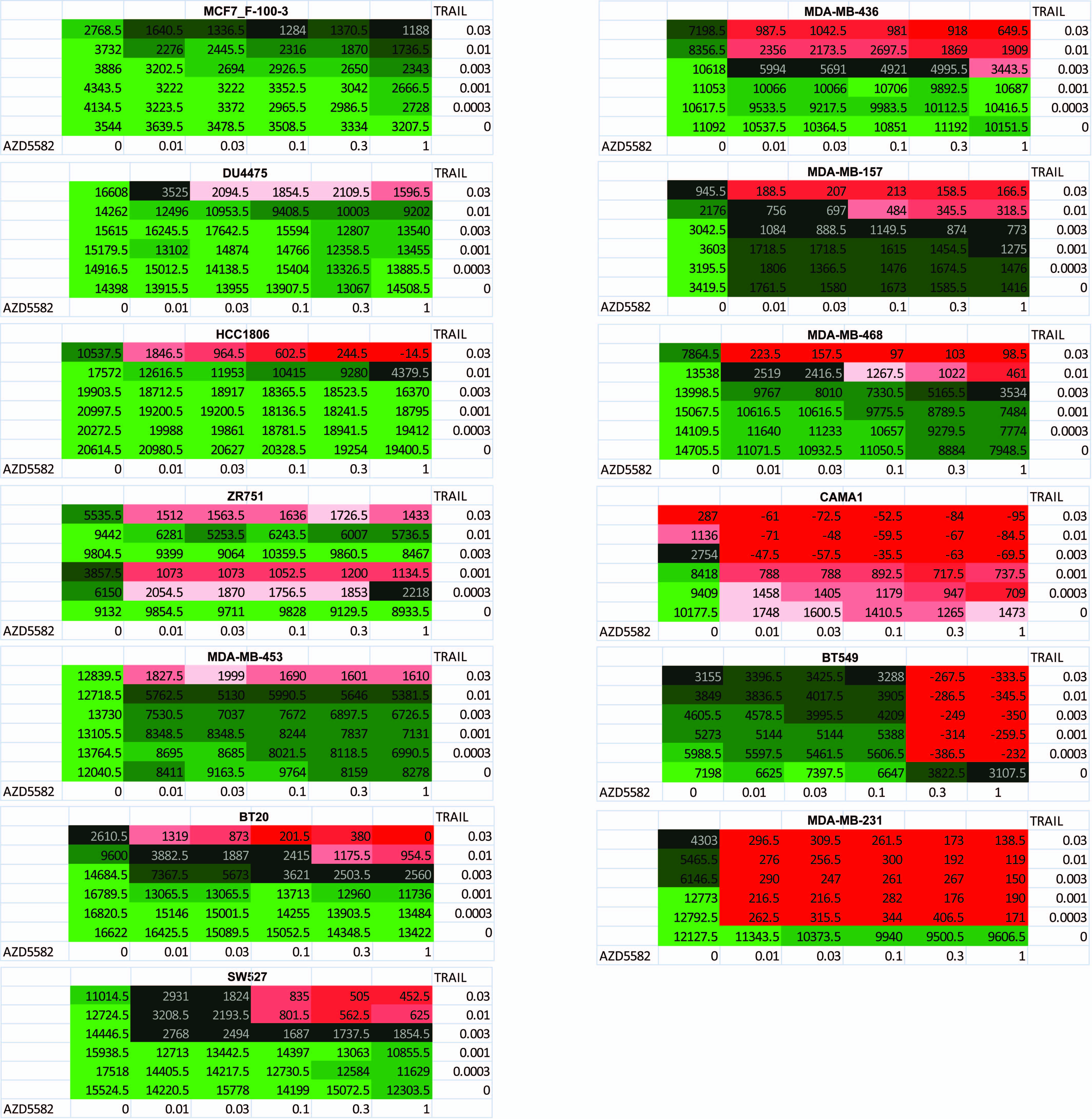


**Supplementary Figure S4. Heat maps from the AZD5582/TRAIL combination screen in breast cancer cell line panel.** Heat maps arranged according to the synergy score represent live cell number upon normalization to Day 0 cell count.


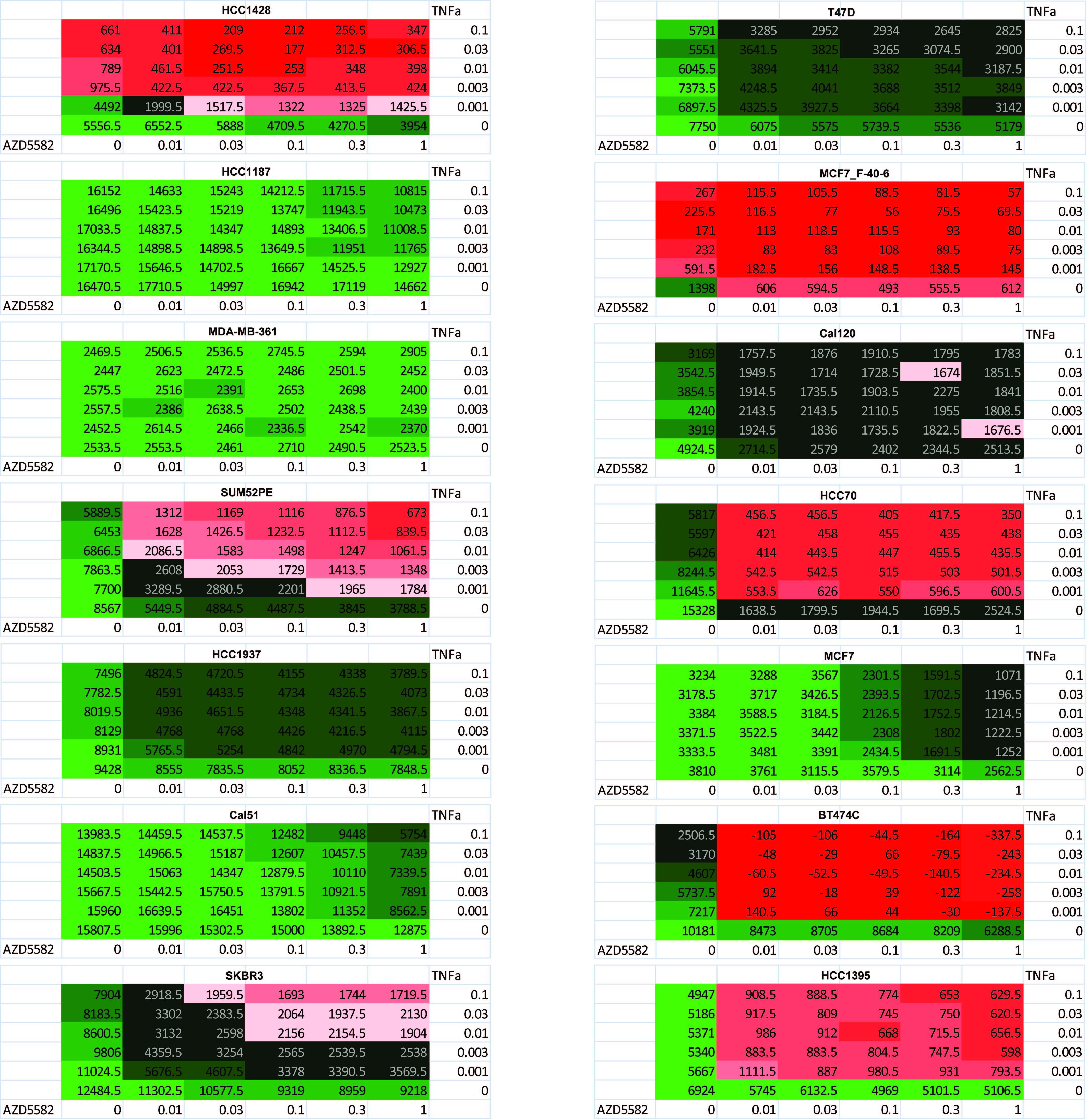


**Supplementary Figure S5. Heat maps from the AZD5582/TNFα combination screen in breast cancer cell line panel accompanying Figure 4.** Heat maps arranged according to the synergy score of AZD5582/TRAIL treatment, represent live cell number upon normalization to Day 0 cell count of cells treated with the AZD5582/TNFα combination.

**Supplementary Figure S6. Dose response curves to AZD5582.** Cell lines were seeded into 384-well plates, 24h later treated with AZD5582 and a proliferation/cell death assay was carried out 24h post-treatment. Error bars represent SEM of means of three independent experiments.

| **Cell line** | **TRAIL+AZD5582**  **synergy score** |
| --- | --- |
| HCA7 | 6.1 |
| HCT116 | 7.7 |
| NCI-H747 | 8.4 |
| Colo741 | 8.6 |
| HT29 | 12.6 |
| Colo205 | 27.4 |
| SW48 | 30.4 |
| SW948 | 31.4 |
| LS174T | 35.5 |
| LS513 | 40.5 |
| CC20 | 48.7 |
| SW620 | 51.8 |
| C32 | 52.4 |
| C75 | 58.2 |
| SW403 | 61.9 |
| SW837 | 61.9 |

**Supplementary Table 1.** AZD5582+TRAIL synergy scores across the panel of colorectal cancer cell lines

| **Protein** | ***p* value** |
| --- | --- |
| DR4 | 0.41 |
| DR5 | 0.42 |
| IAP1 | 0.18 |
| IAP2 | 0.53 |
| XIAP | 0.42 |
| FLIP | 0.18 |
| CASPASE-8 | 0.77 |
| BIM | 0.06 |
| BCL-2 | 0.32 |
| BCL-XL | 0.80 |
| PUMA | 0.07 |
| MCL-1 | 0.07 |
| BAX | 0.28 |
| BAK | 0.41 |
| BID | 0.65 |

**Supplementary Table 2**. Statistical analysis of protein expression levels

| **cell line** | **media** |
| --- | --- |
| Cal51 | 1:1 Mix of DMEM and Ham's F-12 + 10%FCS + 2mM L-Glutamine |
| MDAMB231 | DMEM + 10% FCS + 2mM L-Glutamine |
| KPL1 | DMEM + 10% FCS |
| Sw527 | DMEM + 10%FCS, 2mM Glutamax |
| MDAMB157 | DMEM + 10%FCS |
| MDAMB468 | DMEM + 10%FCS |
| T47D | DMEM + 10%FCS + 2mM L-Glutamine + 1% NEAA |
| BT20 | DMEM + 10%FCS + 10ug/ml Insulin |
| BT20 | DMEM + 10%FCS + 10ug/ml Insulin |
| HS578T | DMEM + 10%FCS + 10ug/ml Insulin |
| Cal148 | DMEM + 20%FBS, 2mM Glutamax + EGF (1ug/100ml) |
| MDAMB453 | DMEM/F12 (1:1) +10% FBS +2mM L-Glutamine |
| CAL120 | DMEM+10% FCS |
| CAMA1 | EMEM + 10%FCS |
| MFM223 | EMEM + 15%FCS + 2mM glutamax + ITS (insulin-transferrin-selentite) |
| EVSAT | EMEM+10%FCS + 25mM HEPES |
| SUM52PE | Ham’s F12 + 5% FCS + 5 ug/ml Insulin + 1 ug/ml Hydrocortisone |
| SUM149PT | Ham's F12+ 5%FCS +HEPES 10mM + Hydrocortisone 1µg/ml + Insulin 5µg/ml |
| MDAMB415 | L-15 + 15% FCS + 2mM L-Glutamine + 10 mcg/ml insulin + 10 mcg/ml glutathione, |
| MDAMB134V1 | L-15 + 20%FCS |
| SKBR3 | McCoy's 5a + 10% FCS |
| KPL4 | RPMI + 10% FCS |
| MDAMB436 | RPMI + 10% FCS + 2mM Glutamine + 10ug/ml Insulin |
| HCC1395 | RPMI + 10% FCS + 2mM L-Glutamine |
| BT474c | RPMI + 10% FCS + 2mM L-Glutamine |
| HCC1187 | RPMI + 10% FCS + 2mM L-Glutamine |
| HCC1806 | RPMI + 10% FCS + 2mM L-Glutamine |
| HCC1937 | RPMI + 10% FCS + 2mM L-Glutamine |
| HCC1954 | RPMI + 10% FCS + 2mM L-Glutamine |
| HCC38 | RPMI + 10% FCS + 2mM L-Glutamine |
| MDAMB361 | RPMI + 10% FCS + 2mM L-Glutamine |
| DU4475 | RPMI + 20% FCS + 2mM L-Glutamine |
| HCC70 | RPMI 1640 + 10%FCS + 2mM glutamax |
| HCC1143 | RPMI 1640 + 10%FCS + 2mM glutamax |
| MCF7 | RPMI 1640 + 10%FCS |
| BT549 | RPMI 1640 + 10%FCS + 2mM L-Glutamine |
| HCC1428 | IMEM (zinc modification) + 10% FCS + 2mM glutamax |
| ZR751 Parental | RPMI 1640 + 10%FCS + 1% sodium pyruvate + 10ug/ml insulin |
| Colo205 | RPMI 1640 + 10%FCS |
| HT29 | EMEM + 10%FCS + 1%NEAA + 1% GLUT |
| Colo741 | RPMI + 10% FCS |
| HCT116 | RPMI + 10% FCS + 2mM L-Glutamine |
| LS174T | EMEM + 5%FCS + 1%NEAA |
| LS513 | RPMI + 10% FCS |
| NCI-H747 | RPMI + 10% FCS |
| SW403 | L-15 + 10% FCS |
| SW620 | L-15 + 10% FCS + 1% sodium pyruvate |
| SW837 | RPMI + 10% FCS |
| SW948 | L-15 + 10% FCS |
| CC20 | DMEM + 10% FCS |
| HCA7 | 1:1 Mix of DMEM and Ham's F-12 + 10%FCS + 2mM L-Glutamine |
| SW48 | RPMI + 10% FCS |
| C32 | IMDM + 10% FCS |
| C75 | IMDM + 10% FCS |

**Supplementary Table 3.** Media formulations

| **Antibody specificity** | **Species** | **Supplier** |
| --- | --- | --- |
| c-IAP-1 | Goat | R&D Systems |
| c-IAP-2 | Mouse | BD Pharmigen |
| XIAP | Mouse | BD Transduction Laboratories |
| BID | Rabbit | Cell Signaling Technology |
| β-Actin | Mouse | Sigma |
| DR4 | Rabbit | Millipore |
| DR5 | Rabbit | Millipore |
| Caspase-8 | Mouse | Santa Cruz Biotechnology |
| PARP | Rabbit | Cell Signaling Technology |
| FLIP | Rabbit | Cell Signaling Technology |
| BIM | Rabbit | Cell Signaling Technology |
| BCL2 | Mouse | Santa Cruz Biotechnology |
| BCL-XL | Rabbit | Cell Signaling Technology |
| NOXA | Rabbit | Cell Signaling Technology |
| PUMA | Rabbit | Cell Signaling Technology |
| BAK | Rabbit | Cell Signaling Technology |
| MCL1 | Rabbit | Cell Signaling Technology |
| BAX | Rabbit | Santa Cruz Biotechnology |
| Anti-goat IgG, HRP-linked | Mouse | Thermo Scientific |
| Anti-rabbit IgG, HRP-linked | Goat | Cell Signaling Technology |
| Anti-mouse IgG, HRP-linked | Horse | Cell Signaling Technology |

**Supplementary Table 4**. Antibodies

| **cell line** | **Seeding density** |
| --- | --- |
| Cal51 | 2000 |
| MDAMB231 | 1000 |
| KPL1 | 1000 |
| Sw527 | 1000 |
| MDAMB157 | 2000 |
| MDAMB468 | 1500 |
| T47D | 1000 |
| BT20 | 1500 |
| HS578T | 1500 |
| Cal148 | 1000 |
| MDAMB453 | 2000 |
| CAL120 | 500 |
| CAMA1 | 2000 |
| MFM223 | 1500 |
| EVSAT | 1000 |
| SUM52PE | 1500 |
| SUM149PT | 1500 |
| MDAMB415 | 2000 |
| MDAMB134V1 | 2500 |
| SKBR3 | 2000 |
| KPL4 | 1000 |
| MDAMB436 | 1500 |
| HCC1395 | 2000 |
| BT474c | 2000 |
| HCC1187 | 2000 |
| HCC1806 | 1000 |
| HCC1937 | 2500 |
| HCC38 | 1000 |
| MDAMB361 | 2500 |
| DU4475 | 2000 |
| HCC70 | 2000 |
| HCC1143 | 1500 |
| MCF7 | 1000 |
| BT549 | 1000 |
| HCC1428 | 2000 |
| ZR751 Parental | 1500 |
| Colo205 | 1500 |
| HT29 | 750 |
| Colo741 | 2000 |
| HCT116 | 750 |
| LS174T | 1000 |
| LS513 | 2000 |
| NCI-H747 | 1500 |
| SW403 | 1500 |
| SW620 | 1000 |
| SW837 | 2000 |
| SW948 | 1000 |
| CC20 | 1500 |
| HCA7 | 1500 |
| SW48 | 1500 |
| C32 | 2000 |
| C75 | 1500 |

**Supplementary Table 5**. Cell line seeding densities
